# A bivalent recombinant protein inactivates HIV-1 by targeting the gp41 prehairpin fusion intermediate induced by CD4 D1D2 domains

**DOI:** 10.1186/1742-4690-9-104

**Published:** 2012-12-07

**Authors:** Lu Lu, Chungen Pan, Yuan Li, Hong Lu, Wu He, Shibo Jiang

**Affiliations:** 1 Key Laboratory of Medical Molecular Virology of Ministries of Education and Health, Shanghai Medical College and Institute of Medical Microbiology, Fudan University, Shanghai 200032, China; 2 Lindsley F. Kimball Research Institute, New York Blood Center, New York, NY 10065, USA; 3 The Institute of Human Virology, Key Laboratory of Tropical Disease Control of MOE, Zhongshan School of Medicine, Sun Yat-Sen University, Guangzhou 510080, China

**Keywords:** HIV-1, gp41, Peptide, Six helix bundle, Inactivation, HIV-1 fusion inhibitor

## Abstract

**Background:**

Most currently approved anti-HIV drugs (*e.g.*, reverse transcriptase inhibitors, protease inhibitors and fusion/entry inhibitors) must act inside or on surface of the target cell to inhibit HIV infection, but none can directly inactivate virions away from cells. Although soluble CD4 (sCD4) can inactivate laboratory-adapted HIV-1 strains, it fails to reduce the viral loads in clinical trials because of its low potency against primary isolates and tendency to enhance HIV-1 infection at low concentration. Thus, it is essential to design a better HIV inactivator with improved potency for developing new anti-HIV therapeutics that can actively attack the virus in the circulation before it attaches to and enter into the target cell.

**Results:**

We engineered a bivalent HIV-1 inactivator, designated 2DLT, by linking the D1D2 domain of CD4 to T1144, the next generation HIV fusion inhibitor, with a 35-mer linker. The D1D2 domain in this soluble 2DLT protein could bind to the CD4-binding site and induce the formation of the gp41 prehairpin fusion-intermediate (PFI), but showed no sCD4-mediated enhancement of HIV-1 infection. The T1144 domain in 2DLT then bound to the exposed PFI, resulting in rapid inactivation of HIV-1 virions in the absence of the target cell. Beside, 2DLT could also inhibit fusion of the virus with the target cell if the virion escapes the first attack of 2DLT.

**Conclusion:**

This bivalent molecule can serve as a dual barrier against HIV infection by first inactivating HIV-1 virions away from cells and then blocking HIV-1 entry on the target cell surface, indicating its potential for development as a new class of anti-HIV drug.

## Background

Thus far, thirty-two anti-HIV drugs (including five fixed-dose combinations) have been licensed by the United States Food and Drug Administration (FDA) for treatment of HIV infection/AIDS ( http://www.hivandhepatitis.com/hiv_and_aids/hiv_treat.html). Most of these drugs act inside the host cell to inhibit viral replication by targeting HIV reverse transcriptase, protease, or integrase. Two of them, enfuvirtide (also known as T20) [1] and Marviroc [2], act on surface of the target cell to block viral fusion and entry by interacting with the HIV-1 gp41 N-terminal heptad repeat (NHR) and the coreceptor, CCR5, respectively. However, none of these anti-HIV drugs can inactivate virions in the absence of the target cell.

Some of the non-ionic surfactants, such as Nonoxynol-9 (N-9), could effectively inactivate HIV-1 virions by lysing the viral envelope membrane [3]. However, because of its high cytotoxicity, it cannot be used as an anti-HIV drug in clinics since it can also damage cellular membranes [4]. Therefore, it is essential to design an HIV inactivator using a protein or peptide that can actively attack the virion before it attaches to and enters into the host cells, with low or no toxic effect on the host cells.

**Figure 1:**
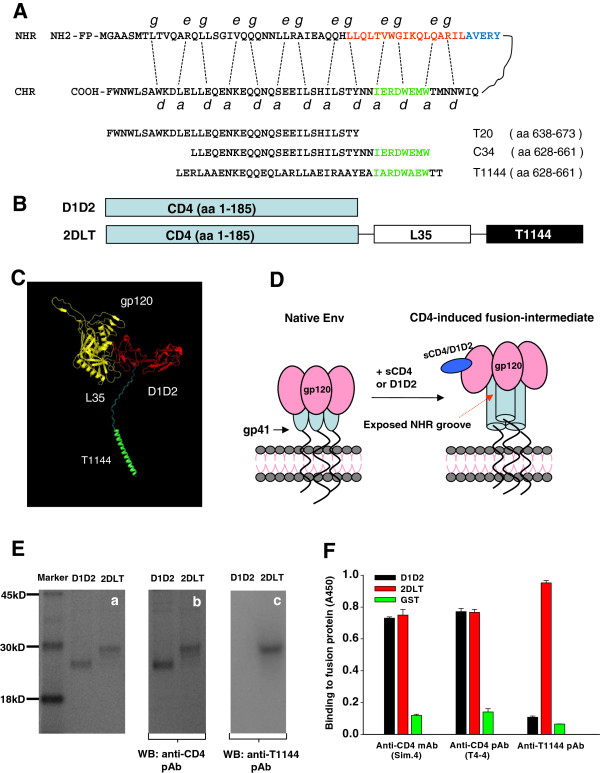
**Design, expression and characterization of 2DLT.A**) The schematic view of the interactions between the NHR and CHR of gp41 and C-peptides. The dashed lines between NHR and CHR indicate the interaction between the residues located at the *e* and *g* positions in the NHR and the *a* and *d* positions in the CHR. The sequences of peptide T20, C34 and T1144 are shown, and the pocket-forming sequence (aa 565–581) in the NHR and pocket-binding domain (aa 628–635) in the CHR are colored in red and green, respectively. **B**) Schematic view of the 2DLT and D1D2 molecules (not drawn to scale). The D1D2 of CD4 (aa 1–185) and T1144 are connected by a 35-mer of linker (GGGGS)_7_. **C**) A diagram showing the predicted structure of 2DLT interacting with gp120. The complex containing the gp120 core (yellow), the two-domain sCD4 (D1D2) (red) and the T1144 (green), was derived from the coordinates of the published X-ray crystallographic complex [11] using the Pymol program ( http://pymol.sourceforge.net). **D**) The model of CD4-induced gp41 PFI. Soluble CD4 (sCD4) or CD4 D1D2 domains bind to HIV-1 Env surface subunit gp120, resulting in the formation of the gp41 PFI with the exposed grooves on the NHR-trimer, which is a target for HIV-1 inactivator. **E**) Characterization of 2DLT. The soluble recombinant proteins 2DLT and D1D2 were expressed in *E. coli* using the PDI-chaperone expression system and analyzed by SDS-PAGE (**a**); Western blot using anti-CD4 polyclonal antibody (**b**); anti-T1144 polyclonal antibody (**c**); and by ELISA using anti-CD4 pAb T4-4, a conformation-dependent mAb Sim.4 and anti-T1144 pAbs **F**). The data are representative of results from three similar experiments performed in triplicate (means ± SD).

To infect a target cell, HIV-1 first binds to the cellular receptor CD4 and then a co-receptor, CXCR4 or CCR5 through its envelope glycoprotein (Env) surface subunit gp120. Subsequently, the HIV-1 Env transmembrane subunit gp41 changes its conformation from native to prehairpin fusion intermediate (PFI) state by inserting the fusion peptide (FP) into the target cell membrane. Three molecules of the gp41 N-terminal heptad repeats (NHR) interact with each other to form a N-trimer, a PFI conformation, which is then associated with the gp41 C-terminal heptad repeats (CHR) to form a hairpin-like six-helix bundle (6-HB) (Figure 1A), bringing the viral and cellular membranes into close proximity necessary for fusion [5-7]. Mutations in the multiple highly conserved tyrosine and dileucine motifs in gp41 cytoplasmic domain lead to a loss of HIV-1 Env-mediated membrane fusion [8], and several specific amino acid changes in gp120 V3 region and gp41 are associated together with CXCR4 and/or CCR5 usage [9], suggesting that both gp120 and gp41 play important roles in HIV-1 entry and are attractive targets for developing an HIV-1 inactivator and/or entry inhibitors [10].

Monomeric soluble CD4 (sCD4) that can specifically binds to the HIV-1 gp120 and then inactivate the virion was one of the first anti-HIV-1 agents tested in clinical trial. Unfortunately, it failed to reduce the viral loads in HIV-1-infected individuals [12,13]. However, sCD4 and CD4-mimetics could efficiently induce the formation of the gp41 PFI with the exposed grooves on the NHR-trimer [14], which is the target of peptidic HIV fusion inhibitors, such as SJ-2176 [15], T20 [16], C34 [17,18] and T1144 [19,20]. These results suggest that a molecule containing a CD4 or CD4-mimetic and a gp41 PFI-binding domain (such as T1144) can inactivate HIV-1 more efficiently than sCD4 or CD4-mimetic since T1144 can bind to the exposed gp41 grooves induced by binding of sCD4 or CD4-mimetic to gp120 to speed the virus inactivation. Based on this hypothesis, we engineered a bivalent protein, designated 2DLT, in which the D1D2 domains of CD4 were linked to T1144 by a 35-mer flexible linker to allow the free movement of the two functional domains in the bivalent molecule (Figure 1B). The D1D2 fragment in this bivalent protein is expected to bind specifically with gp120 on the surface of HIV virions or HIV-infected cells (Figure 1C) and trigger formation of the gp41 PFI with the exposed hydrophobic grooves (Figure 1D), while the T1144 domain can bind to the exposed grooves on the gp41 NHR-trimer, resulting in rapid inactivation of the cell-free HIV-1 before its attachment to the target cell. Indeed, the 2DLT protein could effectively bind to both gp120 and gp41, block gp41 6-HB formation, inactivate cell-free HIV-1 and inhibit HIV-1 Env-mediated cell-cell fusion, but without the sCD4-mediated enhancing effects on HIV-1 infection. Therefore, this engineered bivalent molecule has substantial potential for development as an anti-HIV therapeutic for treatment of patients who fail to respond to the current anti-HIV drugs and as a topical microbicide for preventing sexual transmission of HIV.

## Results

### Construction, expression and characterization of the bivalent fusion protein 2DLT

The expression plasmids pD1D2-PDI and p2DLT-PDI were constructed by linking the DNA fragment encoding D1D2 with those coding the 35-mer linker (GGGGS)_7_ and T1144 sequentially by three-step overlapping PCR using the corresponding primer pairs. The nucleotide sequences of the vectors were confirmed by DNA sequencing. The recombinant bivalent protein 2DLT and the control protein D1D2 (Figure 1B) were expressed in *E. coli.* To avoid the formation of inclusion bodies, we used the protein disulfide isomerase (PDI) chaperone-expression system since we and others have shown that PDI, as a fusion partner, could significantly increase the soluble expression of recombinant proteins in the cytoplasm of *E. coli*[21,22], and we successfully obtained soluble D1D2 and 2DLT proteins. After purification, we analyzed these proteins with SDS-PAGE and Western blot. On the gels, two proteins migrated near the expected position of the proteins (Figure 1Ea, D1D2: ~23KD; 2DLT: ~29KD). Both displayed specific interaction with anti-CD4 polyclonal antibodies (pAb) (Figure 1Eb). While the anti-T1144 pAb bound with 2DLT at the predicted size, no band was revealed in the D1D2 lane (Figure 1Ec). Similarly, anti-CD4 pAb (T4-4) could react with both D1D2 and 2DLT, while anti-T1144 pAb was able to recognize 2DLT only in the ELISA. Notably, both D1D2 and 2DLT could react with the CD4-specific and conformation-dependent monoclonal antibody (mAb) Sim.4 (Figure 1F), suggesting that the soluble D1D2 and 2DLT may have a correctly folded conformation.

### 2DLT inactivated cell-free HIV-1 virions

**Figure 2:**
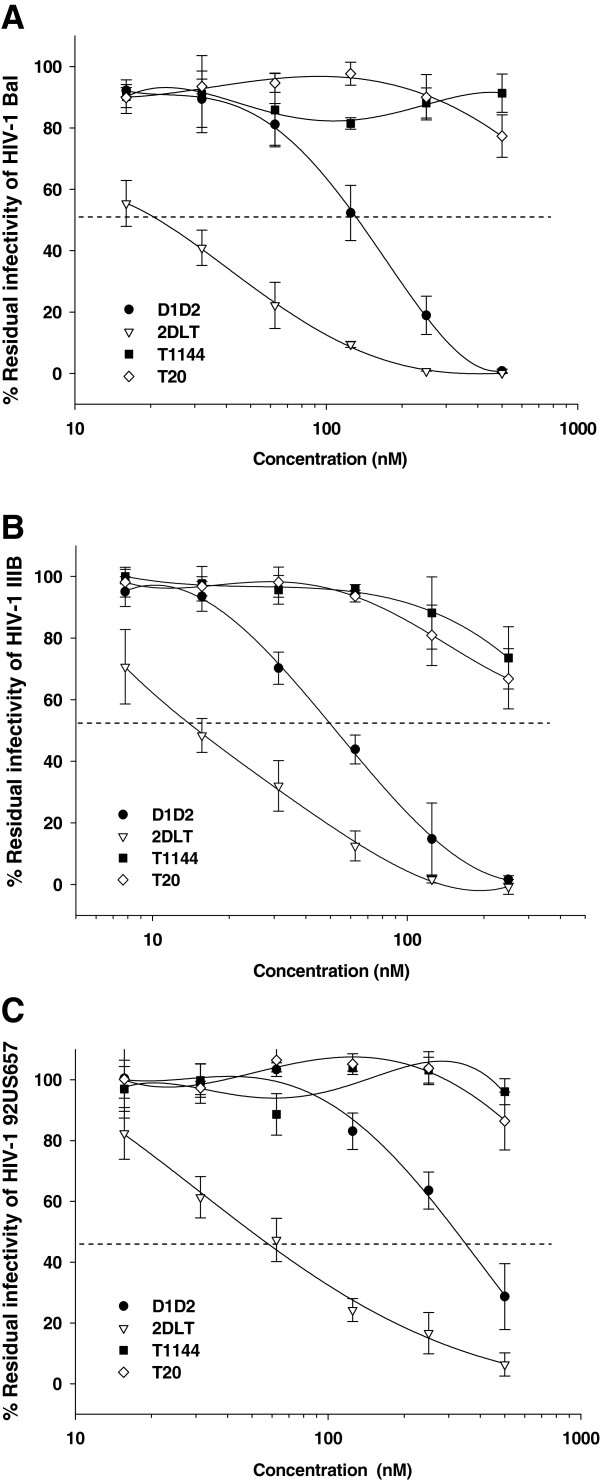
**Inactivation of HIV-1 by 2DLT.** Recombinant proteins and peptides were added to HIV-1 Bal (**A**), HIV-1 IIIB (**B**), and 92US657 (**C**), respectively, followed by incubation for 60 minutes at 4°C. The mixtures were then cooled on ice before addition of PEG-6000 solution at a final concentration of 3% for separation of HIV-1 particles as described in the Methods. The HIV-1 particles containing pellets were re-suspended in tissue culture medium and titered for infectivity. The percentage of residual infectivity is shown in a scale. The data are representative of results from three similar experiments performed in triplicate (means ± SD).

To determine whether 2DLT could inactivate HIV-1 virions, we used PEG-6000 method, as previously described [23-26], to separate HIV-1 from the recombinant proteins 2DLT and D1D2, as well as the control peptides T1144 and T20, and then measured the residual infectivity of the treated HIV-1 particles. As shown in Figure 2 and Table 1, 2DLT inactivated cell-free HIV-1 Bal (laboratory-adapted R5 virus), HIV-1 IIIB (laboratory-adapted X4 virus), and primary R5 isolates with different subtypes (92US657, 93 MW959, and 92TH009) in a dose-dependent manner with 50% effective concentration (EC_50_) ranging from 17.3 to 78.6 nM, which is about 2- to 6-fold more potent than D1D2 against the corresponding viral strains. In contrast, T20 and T1144 at a concentration up to 500 nM did not show significant effects on virus inactivation. Different from the peptide HIV-1 fusion/entry inhibitors T20 and T1144, the bivalent protein 2DLT is capable of directly inactivating HIV-1, making it a good potential candidate for developing novel anti-HIV drugs.

**Table 1:** Inactivation of cell-free HIV-1 R5 and X4 strains by 2DLT

**HIV-1 strain (subtype, tropism)**	**EC**_**50**_**(nM)**			
	**D1D2**	**2DLT**	**T1144**	**T20**
Bal (B, R5)	157.2 ± 34.2	24.5 ± 2.25	>500	>500
IIIB (B, X4)	51.2 ± 9.21	17.3 ± 1.62	>250	>250
92US657 (B, R5)	319.2 ± 56.1	48.7 ± 14.5	>500	>500
93 MW959 (C, R5)	478.2 ± 89.3	78.6 ± 7.04	>500	>500
92TH009 (E/A, R5)	209.5 ± 6.1	69.9 ± 14.4	>500	>500

* Data are representative of two similar experiments tested in triplicate (mean ± SD).

### Like sCD4 and D1D2, 2DLT could bind to gp120 on the cell surface

**Figure 3:**
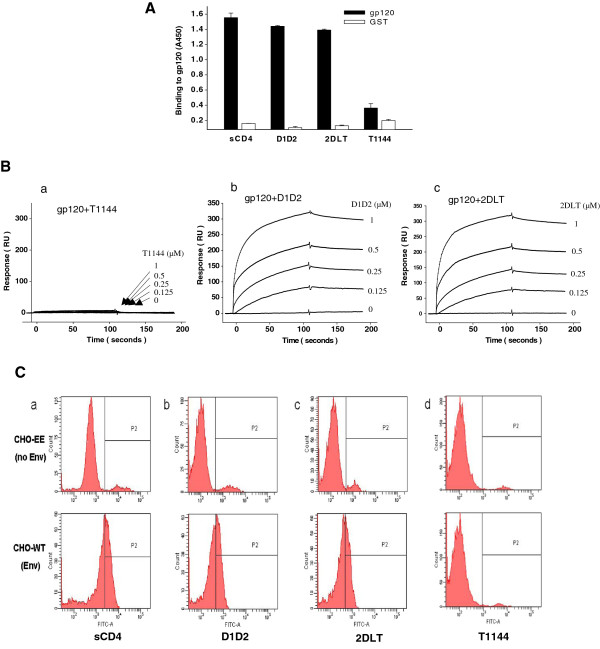
**Binding of the soluble 2DLT and D1D2 proteins to gp120 protein or to gp120 expressed on the cell surface.A**) Binding of sCD4, D1D2, 2DLT, T1144 to rgp120 as measured by ELISA. The data are representative of results from three similar experiments performed in triplicate (means ± SD). **B**) The binding affinity of sCD4 (**a**), D1D2 (**b**), 2DLT (**c**), and T1144 (**d**) to gp120 as determined by SPR assay. The recombinant gp120 (rgp120) was immobilized onto the CM3 sensor chip. The proteins and peptide at various concentrations were injected onto the surface. The affinity constant of each sample was calculated in one-site binding modes by BIAcore evaluation software. **C**) Binding of sCD4, D1D2, 2DLT, and T1144 to the HIV-1 Env (gp120/gp41) expressed on CHO-WT cell surfaces as determined by flow cytometry. CHO-EE cells expressing no HIV-1 Env molecule were used as control cells.

To investigate the inactivation mechanism of 2DLT, we first determined whether the bivalent protein 2DLT retains the biological activities of CD4. As expected, 2DLT could bind to gp120 protein as strongly as sCD4 and D1D2, while T1144 was unable to bind with gp120 as shown in ELISA (Figure 3A). We then used SPR assays to determine whether the 35-mer linker L35 and the C-peptide T1144 in the bivalent protein would affect the binding affinity of the D1D2 domain in 2DLT to gp120. The results confirmed that 2DLT could interact with gp120 in a dose-dependent manner with high binding affinity (KD = 1.9e^-8^) comparable to D1D2 (KD = 2.1e^-8^), while T1144 exhibited no binding to gp120 (Figure 3B). Using a flow cytometry assay, we demonstrated that 2DLT could bind to the native gp120/gp41 (Env) expressed on the surfaces of CHO-WT cells as effectively as sCD4 and D1D2, while T1144 was unable to bind to the cell-expressed Env without sCD4 stimulation. None of these peptides or proteins could bind to CHO-EE cells that express no Env of HIV-1 (Figure 3C).

### Like T1144, 2DLT bound to the groove on the gp41 PFI N-trimer and subsequently blocked gp41 6-HB formation

**Figure 4:**
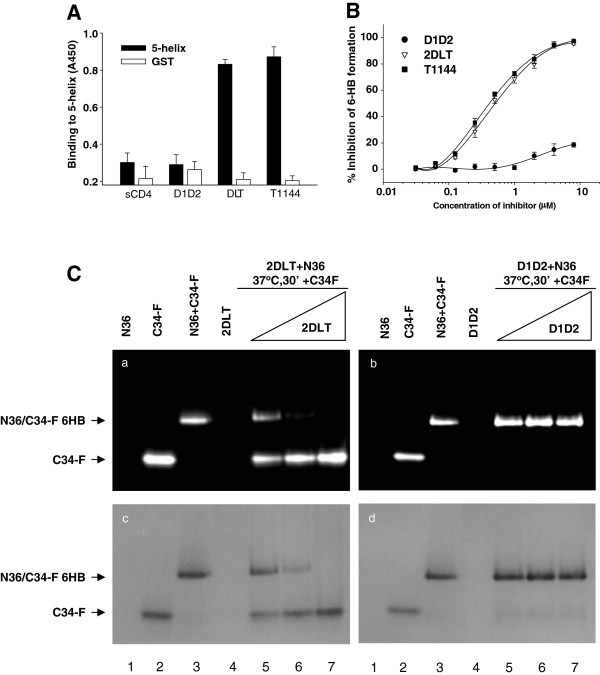
**Inhibition of gp41 6-HB formation by 2DLT.A**) Binding of D1D2, 2DLT, and T1144 to the grooves on the 5-helix as assessed by ELISA. **B**) The inhibition of D1D2, 2DLT, and T1144 to the 6-HB formation between N36 and C34-biotin was detected by ELISA. Each sample was tested in triplicate, and the data are presented as means ± SD. **C**) The inhibition of D1D2 and 2DLT at graded concentration to the 6-HB formation between N36 and C34-FAM was determined by FN-PAGE. The peptide N36 was incubated with 2DLT or D1D2 at 37°C for 30 minutes before addition of the peptide C34-FAM at graded concentrations. After incubation for another 30 minutes, the mixtures were analyzed by FN-PAGE (panels **a** and **b**). The gels in panels a and b were stained with Coomassie Blue (panels **c** and **d**).

To determine whether 2DLT, like T1144, could interact with the exposed grooves on the gp41 N-trimer, we tested the binding activity of 2DLT to 5-helix, a mimic of N-trimer with exposed groove, using an ELISA. As shown in Figure 4A, 2DLT could bind to 5-helix as effectively as T1144, while D1D2 and sCD4 exhibited no significant binding, suggesting that the soluble recombinant protein 2DLT can function like T1144 to interact with the grooves of gp41 N-trimer formed at the PFI state.

The gp41 6-HB formation is a critical step during HIV-1 fusion with the target cell. The peptides derived from the gp41 CHR, *e.g.* C34 and T1144 are able to bind with viral gp41 N-trimer to block the 6-HB core formation [19,27]. Here, we used a sandwich ELISA and fluorescence native polyacrylamide gel electrophoresis (FN-PAGE) to determine if 2DLT, like T1144, possessed inhibitory activity on gp41 6-HB formation in a model system mimicking the gp41 6-HB core formation by mixing the gp41 N36 and C34 (or FAM-labeled C34) peptides at equal molar concentration [17,28]. In the ELISA, 2DLT, like T1144, inhibited the 6-HB formation in a dose-dependent manner with an IC_50_ of 0.5 ±0.06 μM, while D1D2 protein at 10 μM exhibited no significant inhibition (Figure 4B). Similarly, 2DLT could effectively block 6-HB formation in a dose-dependent manner when it was tested at 5, 10, and 20 μM as shown in the FN-PAGE (Figure 4Ca and Cc, lanes 5 to 7), whereas D1D2 protein at the same concentrations showed no significant inhibition (Figure 4Cb and Cd, lines 5 to 7). The D1D2 and 2DLT bands were not observable on the gels because they carry net positive charges, like the N-peptide N36 (lane 1 in Figure 4C) and run in a reversed direction under the native gel condition as previously described [27,29]. These results indicate that 2DLT can interact with the gp41 N-trimer and block the 6-HB core formation between viral gp41 NHR and CHR domains.

### 2DLT could disrupt the function of the CD4-induced gp41 PFI, and caused no significant enhancement of HIV-1 infection in CD4^-^/CCR5^+^ cells

**Figure 5:**
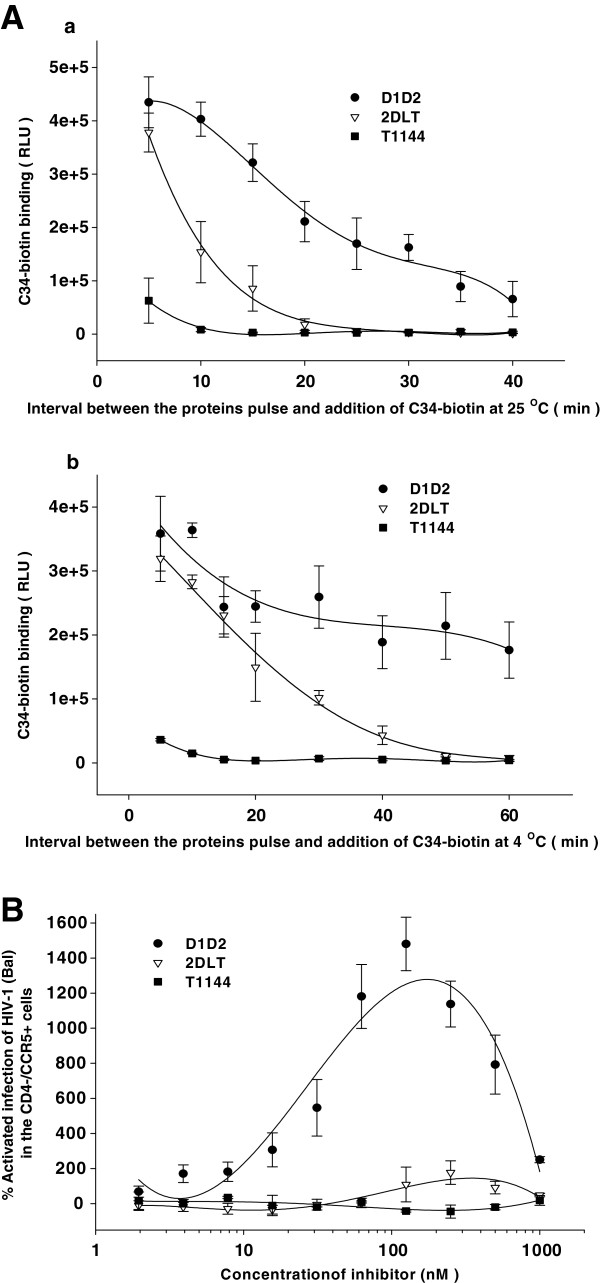
**2DLT-mediated rapid decay of the CD4-induced gp41 PFI and lack of D1D2-induced enhancement of HIV-1 infection in CD4**^**-**^**/CCR5**^**+**^**cells.A**) Decay of the gp41 PFI at 25°C (**a**) and 4°C (**b**) after pulse activation with D1D2 (2.5 μM), 2DLT (2.5 μM) or T1144 (2.5 μM) was measured by a cell-based ELISA, as described in the Methods. **B**) The infectivity of HIV-1 Bal (100 TCID_50_/well) in Cf2Th-CCR5 (CD4^-^/CCR5^+^) cells in the presence of D1D2, 2DLT or T1144 was determined by ELISA for p24 antigen production as described in the Materials and Methods.

Recently, Haim *et al*. have demonstrated that binding of sCD4 or CD4-mimetics to HIV-1 gp120 could induce a short-lived PFI N-trimer, resulting in increased binding of the C34-Ig protein to the groove on the N-trimer, using a cell-based ELISA as described in Methods. Using a similar approach, we examined whether 2DLT may act on this intermediate to rapidly inactive viruses by the interaction of T1144 domain on it and NHR of gp41 on Env. Indeed, binding of C34 to HIV-1 Env was rapidly enhanced after D1D2 pulse, and then gradually decreased as the time of D1D2 pulse was prolonged at 25°C (Figure 5Aa) or 4°C (Figure 5Ab), which are coincident with the report by Haim *et al*. There was no C34 binding immediately after T1144 pulse. Strikingly, the binding ability of C34 to the pulsed Env was lost more rapidly in the 2DLT group than the D1D2 group at 25°C (Figure 5Aa) and 4°C (Figure 5Ab). These results suggest that because of the presence of T1144 in the bivalent molecule, 2DLT can decay the N-trimer-exposed PFI at a faster rate than D1D2. Therefore, we believe that 2DLT may have a mechanism of action different from D1D2 molecule in inactivating the CD4-induced gp41 PFI. The D1D2 domain in 2DLT may destabilize the gp41 PFI triggered by its binding to gp120, while its T1144 domain may interact with the exposed N-trimer in the viral gp41 to form heterogonous 6-HB, resulting in the stabilization of the gp41 PFI. It has been reported that sCD4 can modestly enhance the infectivity of some HIV-1 strains at suboptimal concentrations in CD4^-^CCR5^+^ cells because sCD4 may efficiently replace CD4 on cell surface to drive infection of the CD4-negative and CCR5-positive cells [30]. Here, we investigated whether 2DLT could enhance infectivity of CCR5-using HIV-1 strain Bal in Cf2Th-CCR5 (CD4^-^/CCR5^+^) cells, using D1D2 and T1144 as controls. As shown in Figure 5B, D1D2 at the concentrations from 5 to 200 nM significantly enhanced the infection of the virus in the CD4^-^/CCR5^+^ cells, while 2DLT and T1144 at the same concentrations showed no enhancement of HIV-1 infection, suggesting that 2DLT exhibits no enhancing effect on HIV-1 infectivity in CD4^-^/CCR5^+^ cells.

### 2DLT inhibited HIV-1 infection and HIV-1-mediated cell-cell fusion

**Figure 6:**
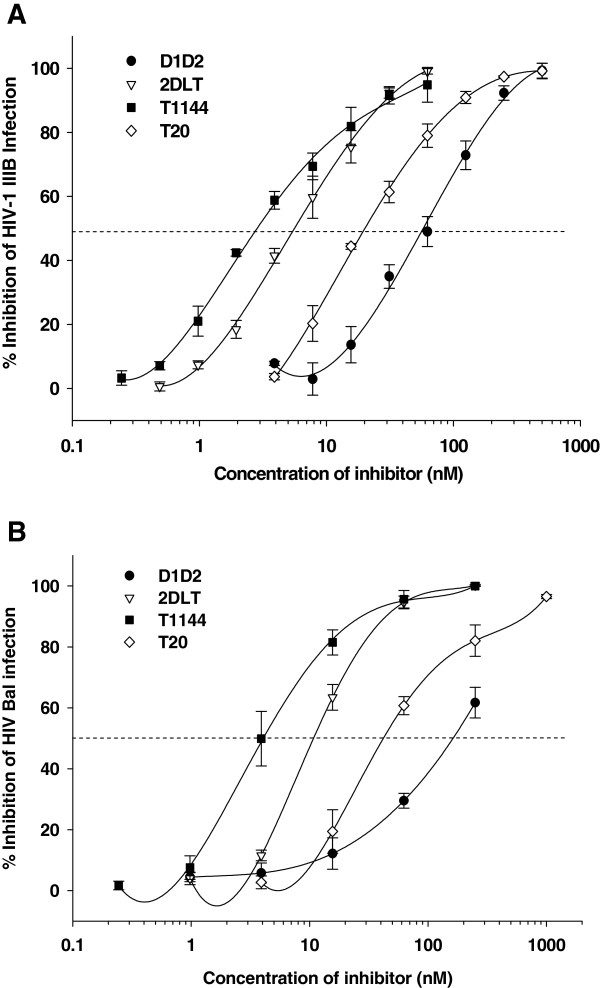
**Inhibition of 2DLT on HIV-1 infection.** The infectivity of IIIB (subtype B, X4) in MT-2 cells (**A**) or HIV-1 Bal (subtype B, R5) in TZM-bl cells (**B**) in the presence or absence of the fusion protein (D1D2 or 2DLT) or peptide (T20 or T1144) was detected by using in-house p24 kits or Promega’s luciferase kits, respectively.

Subsequently, we determined the inhibitory activities of 2DLT against HIV-1 IIIB-mediated cell-cell fusion and against infection by laboratory-adapted HIV-1 IIIB (X4) and Bal (R5) strains in MT-2 and TZM-b1 cells, respectively, as well as several primary HIV-1 isolates in PBMCs using D1D2, T1144 and T20 as controls. Like T1144 and T20, 2DLT also exhibited potent inhibitory activities against HIV-1 IIIB-mediated cell-cell fusion (IC_50_ = 19.03 nM) and infection by HIV-1 strains IIIB (IC_50_ = 5.64 nM) and Bal (IC_50_ = 10.78 nM), which are about 5- to 15-fold more potent than D1D2 (Figure 6). Similarly, 2DLT could also significantly inhibit infection by the primary HIV-1 isolates tested at a low nanomolar level (Table 2). Notably, 2DLT displayed much higher anti-HIV-1 activity than D1D2 in all assays. These results suggest that 2DLT, in addition to its HIV-1 inactivation function, can also serve as an HIV-1 fusion/entry inhibitor, like T20, against a broad spectrum of HIV-1 strains, irrespective of co-receptor usage.

**Table 2:** Inhibitory activity of the recombinant proteins and peptides on HIV-1-mediated cell-cell fusion and HIV-1 replication

**Concentration (nM)**	**D1D2**		**2DLT**		**T20**		**T1144**	
	**IC**_**50**_	**IC**_**90**_	**IC**_**50**_	**IC**_**90**_	**IC**_**50**_	**IC**_**90**_	**IC**_**50**_	**IC**_**90**_
HIV-1 IIIB-mediated cell fusion								
	97.83	137.18	19.03	46.51	20.15	41.71	5.89	15.26
HIV-1 replication								
IIIB (B, X4)	56.80	246.02	5.64	28.79	17.26	117.21	2.13	35.23
Bal (B, R5)	170.20	>250	10.78	44.35	43.35	>250	4.03	28.11
92US657 (B, R5)*	>250	>250	17.17	51.85	56.07	223.92	13.21	42.26
93 MW959 (C, R5)*	>250	>250	18.93	48.86	4.70	18.76	2.35	16.25
92TH009 (E/A, R5)*	25.30	113.69	17.50	71.93	1.43	7.99	1.36	6.21

*Primary HIV-1 isolates.

To determine the effect of preincubation of the virus with an inhibitor on the inhibitor’s anti-HIV-1 activity, we preincubated HIV-1 Bal virus with 2DLT, D1D2, T1144 or T20 for 0, 15, 60 and 240 minutes, respectively, before addition of the mixture to TZM-bl cells. Their inhibitory activity on HIV-1 Bal infection was then tested. We found that the potency of 2DLT remarkably increases with increasing preincubation times. After preincubation for 240 minutes, the inhibitory activity of 2DLT was increased about 4-fold, while that of T1144, D1D2 and T20 was increased only about 0.3- to 0.6-fold (Table 3). This result suggests that preincubation of 2DLT with HIV-1 may trigger the increased exposure of the gp41 PFI, because of the increased binding of D1D2 domain in 2DLT with gp120 on virions, resulting in the increased binding of its T1144 domain to the exposed gp41 PFI and enhanced inhibition of HIV-1 infection.

**Table 3:** Effect of pre-incubation of the virus with inactivators or inhibitors on inhibition of HIV-1 replication

**Preincubation time (min)**	**IC50 ( nM ) for inhibiting HIV-1 Bal infection**			
	**D1D2**	**2DLT**	**T20**	**T1144**
0	137.10 ± 6.66	11.85 ± 2.23	32.35 ± 6.86	2.92 ± 1.01
15	129.25 ± 7.22	7.52 ± 3.65	27.35 ± 3.26	2.71 ± 0.98
60	103.27 ± 6.26	3.22 ± 1.20	20.13 ± 4.21	2.61 ± 0.58
240	86.69 ± 6.13	2.38 ± 0.59	20.51 ± 3.22	2.22 ± 0.82

* Data are representative of two similar experiments tested in triplicate (mean ± SD).

## Discussion

Soluble CD4 (sCD4) was previously recognized as a potential HIV-1 inactivator since it can bind to gp120 for inducing inactivation of HIV-1 virions. However, a very high concentration of sCD4 is required to neutralize infection by primary HIV-1 isolates [14,31,32]*.* Even worse, sCD4 at low concentration can enhance HIV-1 infection of CD4^-^/CCR5^+^ cells [14]. To improve sCD4-mediated HIV-1 neutralizing activity, several groups have constructed hybrid proteins by fusing CD4 or D1D2 with human IgG (CD4-IgG2 or PRO 542) [33,34] or with a monoclonal antibody (mAb) specific for the CD4-induced epitope, such as 17b (sCD4-17b) [35]. In a clinical trial, CD4-IgG2 treatment led to about 0.5 log_10_ reduction in viral load [34]. However, CD4-IgG fusion proteins have two limitations. First, the molecule is too large to be expressed in a large quantity. Second, the inactivator targeting only gp120 may not be very effective in rapid decay of the gp41 PFI, a critical step of inactivating HIV-1 [14]. Therefore, the CD4-induced gp41 PFI is expected to be a novel target for development of HIV inactivators with improved efficacy.

**Figure 7:**
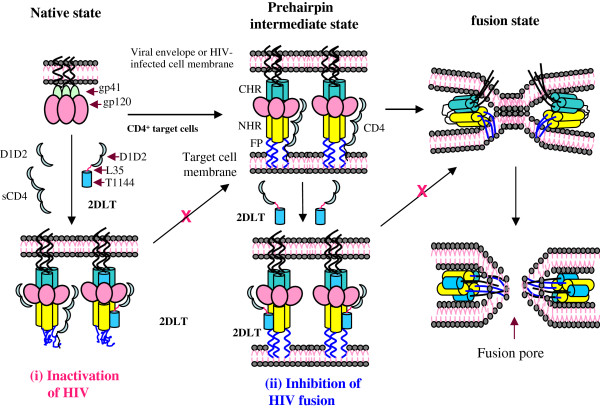
**The putative mechanisms of action of 2DLT to inactivate virions and to inhibit viral fusion and entry.** 2DLT binds to gp120 via its D1D2 domain and induces the exposure of the grooves of the gp41 trimer, which subsequently binds the T1144 domain in 2DLT, leading to irreversible inactivation of HIV-1 (**model a**). Like T1144, 2DLT may also directly bind to gp41 NHR via the T1144 domain to inhibit HIV-1 fusion with the target cell (**model b**).

In this study, we engineered a bivalent HIV-1 inactivator, 2DLT by targeting CD4-induced gp41 PFI with exposed grooves on the NHR-trimer. It consists of a D1D2 fragment of CD4, a 35-mer linker, and a C-peptide T1144 (Figure 1B). As expected, 2DLT could effectively inactivate HIV-1, including both laboratory-adapted and primary strains with different subtypes and tropism (Figure 2 and Table 1). It bound to gp120 via its D1D2 domain (Figure 3A-C) and then interacted with the exposed grooves on the NHR-trimer via its T1144 domain, resulting in a rapid decay of the gp41 PFI (Figure 5A). Unlike sCD4 and D1D2, 2DLT did not enhance HIV-1 infection in CD4^-^/CCR5^+^ cells (Figure 5B). Like T1144, 2DLT could also bind to the exposed groove on the gp41 NHR-trimer (Figure 4A) and inhibit the gp41 6-HB core formation (Figure 4B and C), as well as block HIV-1 fusion and replication through its T1144 domain (Figure 6 and Table 2). The bivalent HIV-1 inactivator 2DLT is able permanently to inactivate HIV-1 at any time it comes into contact with the virions, while T20 can only inhibit HIV-1 fusion within several minutes when the virion or virus-infected cell attaches to the target cell (Figure 7). Compared with the non-ionic surfactant-based virus inactivators, such as N9 [36,37] and C31G [38,39], 2DLT protein has much lower cytotoxicity, with an *in vitro* CC_50_ (50% cytotoxicity concentration) value greater than 100 μM on the cells susceptible to HIV-1 infection and reproductive tract epithelial cells (data not shown), and better specificity since it specifically interacts with the HIV-1 Env, rather than other components that are also present in host cells [40,41]. Compared with sCD4 or D1D2, 2DLT can decay the CD4-induced gp41 PFI much more rapidly and efficiently through the interaction of its T1144 domain with the exposed grooves on the NHR-trimer.

Similar to several gp120-specific mAbs, sCD4 was shown to modestly enhance the infectivity of HIV-1 at suboptimal concentrations in CD4^-^/CCR5^+^ cells [30], which is more prominent in some simian immunodeficiency virus (SIV) and SIV-related HIV-2 strains [14,42-45]. This phenomenon was explained by the hypothesis that sCD4 can efficiently replace cell-surface CD4 to drive virus infection in CD4-negative and CCR5-positive cells [30]. In the present study, we found that the soluble D1D2 molecule could enhance infectivity of a CCR5-using HIV-1 strain Bal in Cf2Th-CCR5 (CD4^-^/CCR5^+^) cells at the concentration from 5 to 200 nM, while 2DLT and T1144 exhibited no enhancement of HIV-1 infection at the same concentration range. This suggests that unlike the soluble D1D2 protein, the D1D2 domains in 2DLT may not be able to replace CD4 on cell surface to drive HIV-1 infection in the CD4^-^/CCR5^+^ cells or the rapid decay of the gp41 fusion-intermediate mediated by 2DLT results in the abolishment of the potential enhancement of HIV-1 infection.

We have demonstrated that like the isolated T1144 peptide, the T1144 domain in 2DLT could also effectively inhibit HIV-1 infection and HIV-1-mediated cell-cell fusion (Figure 6 and Table 2). The dual functions of 2DLT make it superior to the other kind of inactivators (such as N9 or C31G) since if the HIV-1 virions are not completely inactivated, 2DLT is able to block the viral fusion and entry with its T1144 domain.

We have not tested the ability of 2DLT to induce drug-resistant mutations in HIV-1 variants because it will be difficult to interpret the results from the HIV entry inhibitor-induced mutation studies. For example, people may generally believe that sCD4 should induce drug-resistant mutations in the CD4-binding site in viral gp120. However, the mutation sites in the sCD4-resistant HIV-1 variants were mainly located in the V1, V2, and V3 loops in gp120 and the NHR region in gp41 [46]. Similarly, the mutation sites in the HIV-1 variants resistant to T2635, an analogous peptide of T1144, were not located in the hydrophobic pocket of the gp41 NHR domain, which is the main target site of T2635, T1144 and other C-peptides with the pocket-binding domain, but rather in other regions in gp120 and gp41 [47]. This could be possibly explained by the fact that both the CD4 binding site in gp120 and the hydrophobic pocket in gp41 are highly conserved, and the viruses with mutations at these sites may not survive due to the loss of the critical viral functions. Therefore, both D1D2 and T1144 may possess high genetic barrier to resistance. We expect that 2DLT may have a higher genetic barrier to resistance than D1D2 and T1144 when each of them is used alone since 2DLT, like the combination of D1D2 and T1144, is able to simultaneously interact with two target sites, while D1D2 or T1144 can bind to only one target site.

## Conclusions

We have designed and engineered a bivalent protein 2DLT, which can speed the T1144 domain-mediated decay of the gp41 PFI induced by binding of D1D2 domain to gp120, resulting in rapid inactivation of HIV-1 virions. It may also inhibit HIV-1 fusion and entry through its T1144 domain in case when the HIV-1 virion escapes from 2DLT-mediated inactivation. Both of its binding sites in gp120 and gp41 are highly conserved, and it can actively attack the virus in the absence of host cells, making it a promising candidate for further development as a therapeutic for the treatment of HIV/AIDS or as a topical microbicide for preventing sexual transmission of HIV.

## Methods

### Materials

CHO cells stably transfected with either the HIV-1_HXB2_ Env-expressing vector pEE14 (CHO-WT) or control pEE14 vector (CHO-EE) were cultured in Glutamine-deficient minimal essential medium (GMEM-S) containing 400 μM Methionine sulfoximine (Sigma, St. Louis, MO). Cf2Th/syn CCR5 cells stably expressing CCR5 receptors were cultured in DMEM complemented medium with 10% FBS, pen/strep, 500 μg/ml G418, 500 μg/ml zeocin and 3 μg/ml puromycin (Invitrogen, Carlsbad, CA). MT-2 and TZM-b1 cells, as well as laboratory-adapted HIV-1 strains IIIB and Bal, and primary HIV-1 isolates were obtained from the AIDS Research and Reference Reagent Program of NIH. The N-peptide N36 (aa 546–581), and C-peptides C34 (aa 628–661), T1144, and T20 (aa 638–673), used in this study were derived from the NHR and CHR, respectively, of the HIV-1_HXB2_ gp41 (Figure 1A). These peptides (> 95% purity) were synthesized by a standard solid-phase FMOC method using an Applied Biosystems model 433A peptide synthesizer.

### Construction of vectors encoding D1D2 and 2DLT

To create the expression plasmid pD1D2-PDI and p2DLT-PDI, DNA fragments encoding D1D2 (aa 1–185 of CD4), the 35-mer linker (GGGGS)_7_, and T1144 were linked together by three-step overlapping PCR. We took p2DLT-PDI as an example. First, the D1D2 (FD1D2his:   5^′^-CGCGGATCCCATCACCATCACCATCATAAGAAAGTGGTGCTG-3^′^,  RD1D2:  5^′^-CACTTCCTCCTCCTCTATGCTGGAGGCCTTCTGGAA-3^′^),  L35  (FL35:  5^′^-GGAGGAGGA GGAAGTGGCGGCGGCGGCTCGGGTGGTGGTGGTTCTGGAGGTGGCGGTAGCGGAGGTGGAGGTAGTGGAGGC-3^′^,  RL35:  5^′^-GCTACCTCCGCCTCCCGAACCTCCGCCTCCA CTACCTCCACCTCCGCTACCGCCACCTCCAGAACCACCACCACCCGAG-3^′^) and T1144 (FT1144: 5^′^-GAGGCGGAGGTAGCACGACCTGGGAAGCATGGGACAGAGCTATTGCTG AATACGCAGCTAGGATAGAAGCTTTACTCAGAGCTTTA-3^′^,  RT1144:  5^′^-CGGAGAT CTCTATAATTCCCTTAAGGCTGCTTCATTCTTTTCTTGCTGTTCTTGTAAAGCTCTGAGTAAAGCTTCTATCC-3^′^)  DNA  fragments  were  genera-ted by overlapping PCR using the corresponding primer pairs. Second, the DNA fragments coding for L35 and T1144 were linked by overlapping PCR with the primers FL35 and RT1144. Third, the two DNA fragments encoding D1D2 and L35-T1144 were linked by overlapping PCR with the DNA fragment D1D2 and the primers FD1D2his and RT1144. Finally, the amplified DNA fragment coding for 2DLT was digested by *BamHI* and *EcoR I* and inserted into the expression vector pGEX-6p-1 to generate the p2DLT plasmid. In order to prevent the formation of the inclusion bodies in *E. coli*, we inserted a protein disulfide isomerase (PDI) [21,22] DNA sequence (aa 18–508) with PreScission protease cutting site (called ppase site) in the N terminus into the EcoR I and Xho I sites located at the C terminus of His-2DLT gene in the plasmid p2DLT to extend the GST-his-2DLT reading frame, resulting in the generation of chimeric GST-his-2DLT-ppase-PDI. This plasmid is called p2DLT-PDI. The sequences were confirmed by DNA sequencing.

### Protein expression and purification

To express D1D2 and 2DLT fusion proteins, *Escherichia coli* strain Rosetta 2 (DE3) pLysS (Novagen) was transformed with pD1D2-PDI and p2DLT-PDI, respectively, cultured at 37°C to OD600 = 0.4, then induced at 16-22°C for 8–12 h with 0.4 mM IPTG. The cells were harvested and lysed by sonication in the presence of protease inhibitor mixture (Roche). After centrifugation, supernatants containing the fusion protein were collected. The protein was purified with Glutathione-Sepharose 4B affinity columns and cleaved with PreScission^TM^ Protease (GE Healthcare). These fusion proteins were purified by His·Bind® Purification Kit (Novagen) and fast protein liquid chromatography (FPLC), and then analyzed by SDS-PAGE.

### SDS-PAGE and Western blot analysis

Purified fusion proteins were analyzed by SDS-PAGE as previously described [48]. Briefly, D1D2 or 2DLT was mixed with 4X SDS sample buffer (Novagen, Gibbstown, NJ) and boiled for 5 minutes or kept at room temperature (RT) before loading onto a 10-20% Tricine-Glycine gel (Invitrogen, Carlsbad, CA). The electrophoresis was conducted in SDS-PAGE running buffer with 125 V constant voltage at 4°C. In Western blot, the anti-human CD4 and anti-T1144 polyclonal antibodies were used.

### Enzyme-linked immunosorbent assay (ELISA)

D1D2 and 2DLT fusion proteins were characterized by ELISA as previously described [49]. Briefly, they were coated onto a 96-well polystyrene plate (Costar, Corning Inc., Corning, NY) (10 μg/ml in 0.1 M Tris–HCl, pH 8.8), which was blocked with 2% non-fat milk in PBS. The polyclonal antibodies against CD4, conformation-dependent monoclonal antibody against CD4 (Sim.4) and anti-T1144 polyclonal antibody, respectively, were added to the plate. After incubation at 37°C for 60 min, horseradish peroxidase (HRP)-labeled antibodies (ZYMED Laboratories, S. San Francisco, CA) and the substrate TMB (Sigma) were added, sequentially. The binding of D1D2 and 2DLT to gp120 or gp41 NHR, and their inhibitory activity on gp41 6-HB formation were determined by ELISA as previously described [50].

### Flow cytometry

The binding of D1D2 and 2DLT to gp120/gp41 expressed on the cell surface was detected by flow cytometry as previously described [49,51]. Briefly, the cultured CHO-WT (with Env) and CHO-EE (with no Env) cells were detached from plate and washed with wash buffer (PBS containing 5% GBS) three times and incubated with the testing protein for 1 h at 4°C. After three washes, anti-CD4 or anti-T1144 polyclonal antibody was added for 1 h at 4°C. After three washes, FITC-conjugated anti-rabbit or mouse antibody was added and incubated for 1 h at 4°C. After three washes, the cells were examined by flow cytometry and the fluorescence intensity was recorded by FACSCalibur (Becton Dickinson).

### Surface plasmon resonance (SPR) assay

The binding affinity of D1D2 and 2DLT to gp120 was measured by SPR using the BIAcore3000 system (Pharmacia, Piscataway, NJ), following the Manual of the Biomolecular Interaction Analysis (BIA) Technology as described previously [52]. Briefly, gp120 (100 μg/ml) was immobilized onto the CM3 sensor chip by amine coupling, and the unreacted sites were blocked with ethanolamine. The dissociation reaction was done by washing with running buffer (10 mM HEPES pH7.4 containing 0.15 M NaCl, 3.4 mM EDTA and 0.005% v/v surfactant) for at least 2 min.

### Fluorescence native polyacrylamide gel electrophoresis (FN-PAGE)

FN-PAGE for detecting 6-HB formation as described before [27]. Briefly, a testing peptide or protein (100 μM) was pre-incubated with N36 (100 μM) at 37°C for 30 min, followed by addition of C34-FAM (100 μM) at 37°C for 30 minutes. The mixtures were added into Tris-glycine native sample buffer (Invitrogen, Carlsbad, CA). The samples (20 μl) were then loaded onto Tris-glycine gels (18%; Invitrogen, Carlsbad, CA), which were run under 120 V constant voltage at room temperature for 1 h. The gels were stained and visualized with the FluorChem 8800 Imaging System (Alpha Innotech Corp., San Leandro, CA) using a transillumination UV light source with excitation wavelength at 520 nm and then with Coomassie Blue.

### Inhibition of HIV-1 infection

Inhibitory activities of D1D2 and 2DLT on HIV-1 infection were determined as previously described [53,54]. For inhibition of HIV-1 IIIB (subtype B, X4) infection, 100 TCID_50_ of the virus was added to 1 × 10^4^/ml MT-2 cells in RPMI medium 1640 containing 10% FBS in the presence or absence of the test peptide or protein overnight. The culture supernatants were removed, and fresh media were added. On the fourth day post-infection, culture supernatants were collected for detection of p24 antigen by ELISA. For inhibition of infection by the HIV-1 strain Bal (subtype B, R5), TZM-bl cells (1 × 10^5^/ml) were pre-cultured overnight and infected with Bal at 100 TCID_50_ in the presence or absence of the test peptide or protein overnight. The cells were harvested and lysed on the fourth day post-infection with lysing reagent. The luciferase activity was analyzed using a luciferase kit (Promega, Madison, WI) and a luminometer (Ultra 386, Tecan, Durham, NC) according to the manufacturer’s instructions. For testing the effect of pre-incubation times on the inhibitory activity of 2DLT, the TZM-b1 assay was performed after pre-incubation of the HIV-1 Bal virus with the inhibitors for 0, 15, 60 and 240 min. For inhibition of primary HIV-1 isolate infection, peripheral blood mononuclear cells (PBMCs) were isolated from the blood of healthy donors. The PHA-stimulated cells were infected with a primary HIV-1 isolate at a multiplicity of infection (MOI) of 0.01 in the absence or presence of peptide or protein at graded concentrations. The supernatants were collected on the 7th day post-infection and tested for p24 antigen by ELISA as previously described [54,55]. The percent inhibition of p24 production or luciferase activity was calculated.

### Inactivation of HIV-1 virions

The virus inactivation by D1D2 and 2DLT was determined as previously described [25,56,57]. Briefly, 100 μl of the protein or peptide at graded concentration were added to 100 μl of an HIV-1 strain (200 TCID_50_/ml), followed by incubation at 4°C for 1 h. Then, PEG-6000 was added to the treated virus at final concentration of 3%, 4°C for 1 h. The mixture was centrifuged on a microfuge at 15,000 rpm for 30 min. The supernatants were removed and the pellet was washed with 3% PEG in PBS containing 10 mg/ml BSA. The viral pellet was then resuspended in 100 μl of PBS, before addition of 100 μl MT-2 or TZM-bl cells (1 × 10^5^/ml). After cultur at 37°C for 3 days, p24 production in MT-2 cell culture or luciferase activity in TZM-bl cell culture was tested as previously described [58-60].

### Cell-based ELISA

To measure the effects of D1D2 and 2DLT on induction of short-lived PFI of HIV-1 Env, we used a cell-based ELISA as previously described [14]. Briefly, CHO-WT cells steadily expressing HIV-1 Env were seeded in 96-well plates (5 × 10^4^/well). Cells were then harvested and washed twice with blocking buffer (35 mg/ml BSA, 10 mg/ml non-fat dry milk, 1.8 mM CaCl_2_, 1 mM MgCl_2_, 25 mM Tris, pH 7.5 and 140 mM NaCl). For pulse activation experiments, the cells were incubated with D1D2 (2.5 μM) or 2DLT (2.5 μM) suspended in blocking buffer for three minutes, washed three times with blocking buffer and incubated with the C34-biotin (2 μM). To study the temperature dependence of NHR groove exposure, the D1D2- or 2DLT-pulsed cells were incubated at the requisite temperature for different lengths of time. The cells were subsequently returned to room temperature for incubation with C34-biotin. Cells were then washed four times with blocking buffer and four times with washing buffer (140 mM NaCl, 1.8 mM CaCl_2_, 1 mM MgCl_2_ and 20 mM Tris, pH 7.5). A horseradish peroxidase-conjugated streptavidin (ZYMED Laboratories, S. San Francisco, CA) was then incubated with the samples for 45 minutes at room temperature. Cells were washed 5 times with blocking buffer and 5 times with washing buffer. HRP enzyme activity was determined after the addition of 33 μl per well of a 1:1 mixture of Western Lightning oxidizing and luminal reagents (Perkin Elmer Life Sciences) supplemented with 150 mM NaCl. Light emission was measured.

### HIV-1 infectivity in CD4^-^/CCR5^+^ cells treated by D1D2 or 2DLT

The effects of D1D2 and 2DLT on HIV-1 infection of CD4^-^/CCR5^+^ target cells were evaluated as previously described [14]. Briefly, HIV-1 Bal (100 TCID_50_/well was cultured with Cf2Th-CCR5 cells (1 × 10^6^ cells per well) at room temperature for 1 h, then was resuspended, followed by addition of D1D2 or 2DLT at different concentrations. After a short centrifugation, the mixture was incubated for 8–12 h at room temperature. The viral infectivity was measured three days later.

### Inhibition of HIV-1-mediated cell-cell fusion

HIV-1 mediated cell-cell fusion was measured with a dye transfer assay as previously described [27,29]. In brief, the HIV-1_IIIB_ chronically infected H9 (H9/HIV-1_IIIB_) cells were labeled with Calcein-Am (Molecular Probes, Inc., Eugene, OR). After washes, the fluorescence-labeled H9/HIV-1_IIIB_ cells were incubated with MT-2 cells at 37°C for 2 h in the absence or presence of an inhibitor at a graded concentration. The percentage of fused cells was counted under a fluorescence microscope (Zeiss, Germany), and the 50% inhibitory concentration of each drug was calculated with the Calcusyn software program [27,29].

## Abbreviations

CHR: C-terminal heptad repeat; NHR: N-terminal heptad repeat; HR: Heptad repeat; PFI: Prehairpin fusion-intermediate; Native-PAGE: Native polyacrylamide gel electrophoresis; 6-HB: Six-helix bundle; sCD4: Soluble CD4; CC_50_: 50% cytotoxicity concentrations.

## Competing interests

The authors declare that they have no competing interests.

## Authors’ contributions

SJ conceived the idea and designed research. LL, CP, YL, HL, and WH performed research. LL, and SJ analyzed the data and wrote the paper. All authors read and approved the final manuscript.
